# Leucine‐rich repeat kinase 2 interacts with p21‐activated kinase 6 to control neurite complexity in mammalian brain

**DOI:** 10.1111/jnc.13369

**Published:** 2015-10-19

**Authors:** Laura Civiero, Maria Daniela Cirnaru, Alexandra Beilina, Umberto Rodella, Isabella Russo, Elisa Belluzzi, Evy Lobbestael, Lauran Reyniers, Geshanthi Hondhamuni, Patrick A. Lewis, Chris Van den Haute, Veerle Baekelandt, Rina Bandopadhyay, Luigi Bubacco, Giovanni Piccoli, Mark R. Cookson, Jean‐Marc Taymans, Elisa Greggio

**Affiliations:** ^1^ Department of BiologyUniversity of PadovaPadovaItaly; ^2^ San Raffaele Science Park and Università Vita‐Salute San RaffaeleMilanoItaly; ^3^ Laboratory of NeurogeneticsNational Institute on Aging/NIHBethesdaMarylandUSA; ^4^ Laboratory for Neurobiology and Gene TherapyKU LeuvenLeuvenBelgium; ^5^ Department of Molecular Neuroscience UCLReta Lila Weston Institute of Neurological StudiesInstitute of NeurologyLondonUK; ^6^ School of PharmacyUniversity of ReadingReadingUK; ^7^ Department of Molecular NeuroscienceUCL Institute of NeurologyQueen SquareLondonUK; ^8^ Leuven Viral Vector CoreKU LeuvenLeuvenBelgium; ^9^ Present address: Jean‐Pierre Aubert Research CenterUMR837rue Polonovski ‐ 1 place de VerdunLille59045France

**Keywords:** LRRK2, neurodegeneration, neuronal cyto‐skeleton, p21‐activated kinases, Parkinson's disease

## Abstract

Leucine‐rich repeat kinase 2 (*LRRK2*) is a causative gene for Parkinson's disease, but the physiological function and the mechanism(s) by which the cellular activity of LRRK2 is regulated are poorly understood. Here, we identified p21‐activated kinase 6 (PAK6) as a novel interactor of the GTPase/ROC domain of LRRK2. p21‐activated kinases are serine‐threonine kinases that serve as targets for the small GTP binding proteins Cdc42 and Rac1 and have been implicated in different morphogenetic processes through remodeling of the actin cytoskeleton such as synapse formation and neuritogenesis. Using an *in vivo* neuromorphology assay, we show that PAK6 is a positive regulator of neurite outgrowth and that LRRK2 is required for this function. Analyses of post‐mortem brain tissue from idiopathic and LRRK2 G2019S carriers reveal an increase in PAK6 activation state, whereas knock‐out LRRK2 mice display reduced PAK6 activation and phosphorylation of PAK6 substrates. Taken together, these results support a critical role of LRRK2 GTPase domain in cytoskeletal dynamics *in vivo* through the novel interactor PAK6, and provide a valuable platform to unravel the mechanism underlying LRRK2‐mediated pathophysiology.

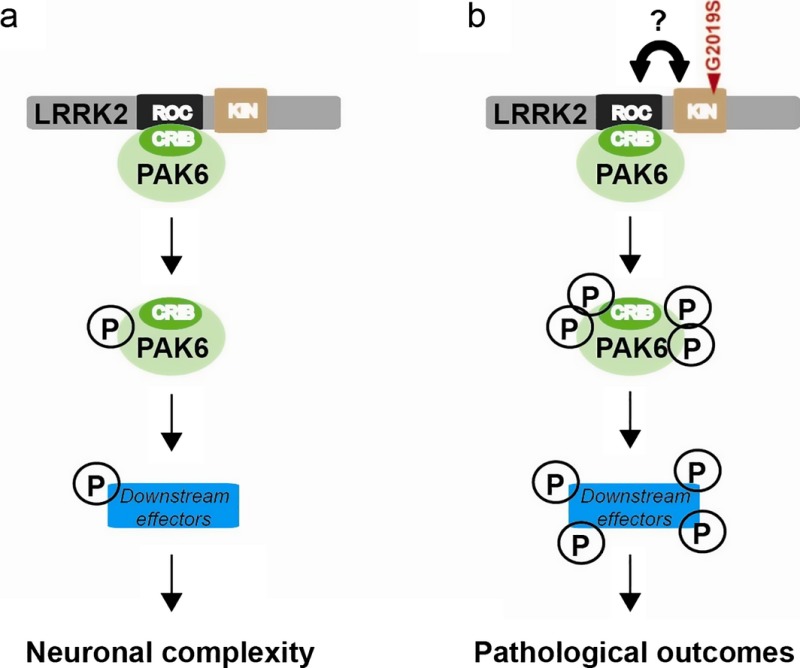

We propose p21‐activated kinase 6 (PAK6) as a novel interactor of leucine‐rich repeat kinase 2 (LRRK2), a kinase involved in Parkinson's disease (PD). In health, PAK6 regulates neurite complexity in the brain and LRRK2 is required for its function, (a) whereas PAK6 is aberrantly activated in LRRK2‐linked PD brain (b) suggesting that LRRK2 toxicity is mediated by PAK6.

**Abbreviations used**
COR: C‐terminus Of ROC
CRIB: Cdc42/Rac‐interactive binding
DAPK1: death‐associated protein kinase 1
eGFP: enhanced green fluorescent protein
EV: empty vector
GAK: cyclin G‐associated kinase
iPD: idiopathic PD
IR: immunoreactivity
LIMK1: LIM domain kinase 1
LRRK1: leucine‐rich repeat kinase 1
LRRK2: leucine‐rich repeat kinase 2
LV: lentiviral vector
MASL1: malignant fibrous histiocytoma‐amplified sequences with leucine‐rich tandem repeats 1
PAK6: p21‐activated kinase 6
PAKs: p21‐activated kinases
PD: Parkinson's disease
rAAV: recombinant adeno‐associated viral
ROC: ras of complex

ROCO proteins constitute a family of proteins with a Ras‐like domain, termed ras of complex (ROC), which is always followed by a C‐terminus Of ROC (COR) domain of unclear function (Bosgraaf and Van Haastert 2003). Humans possess four ROCOs, namely leucine‐rich repeat kinase 1 (LRRK1), leucine‐rich repeat kinase 2 (LRRK2), death‐associated protein kinase 1 (DAPK1), and malignant fibrous histiocytoma‐amplified sequences with leucine‐rich tandem repeats 1 (MASL1) (Lewis 2009; Civiero *et al*. 2014). Human ROCOs are capable of binding guanine nucleotides *via* their ROC domain and nucleotide binding seems important for complex formation and kinase activity (Lewis *et al*. 2007; Jebelli *et al*. 2012; Biosa *et al*. 2013; Dihanich *et al*. 2013), suggesting that this domain is a central hub in ROCO function. Although ROCO proteins have been formally described over 10 years ago, their cellular functions remain elusive as well as the mechanisms by which these proteins, together with other signaling molecules, regulate cellular processes.

Special interest has been directed at understanding the cellular functions of LRRK2, given that mutations in this gene are a common cause of Parkinson's disease (PD) (Paisan‐Ruiz *et al*. 2004, 2008; Zimprich *et al*. 2004). LRRK2 has been linked with several pathways relevant for neuronal physiology, including autophagy (Plowey *et al*. 2008; Gomez‐Suaga *et al*. 2012; Manzoni *et al*. 2013), vesicle trafficking (Piccoli *et al*. 2011; MacLeod *et al*. 2013; Cirnaru *et al*. 2014), neurite outgrowth (MacLeod *et al*. 2006; Dachsel *et al*. 2010; Winner *et al*. 2011), cytoskeletal dynamics (Kett *et al*. 2011; Caesar *et al*. 2013, 2015; Habig *et al*. 2013; Law *et al*. 2013), and inflammation (reviewed in Russo *et al*. 2014). Although some of these functions may appear unrelated, they all rely on the presence of a functional cytoskeleton. Vesicles traffic *via* the cytoskeleton, neurite growth is dynamically balanced between the opposing actions of microtubules and F‐actin, and activated macrophages migrate *via* filopodia and membrane blebs (Ma and Baumgartner 2013).

LRRK2 is a large and complex molecule that contains serine‐threonine kinase and GTPase activities (Greggio 2012; Taymans 2012). Kinase activity has been intensively studied, as there is great interest in identifying therapies for PD and kinases are ideal targets. To date, a number of LRRK2 putative substrates have been identified (Matta *et al*. 2012; Yun *et al*. 2013; Martin *et al*. 2014) but the most consistently reported is LRRK2 itself. LRRK2 undergoes autophosphorylation *in vitro* (Greggio and Cookson 2009) and *in vivo* (Sheng *et al*. 2012), possibly acting as an intramolecular regulator of ROC by phosphorylating serine‐threonine residues important for nucleotide binding (Greggio *et al*. 2009; Webber *et al*. 2011; Greggio 2012; Taymans 2012), thus positioning ROC as the signaling output of LRRK2 activity. However, heterologous effectors of ROC similar to Ras effector kinases for Ras GTPases have not yet been reported.

Here, starting from an unbiased protein array screen we identified p21‐activated kinase 6 (PAK6) as a potential binding partner for LRRK2 (Beilina *et al*. 2014). We found that the GTPase/ROC domain of LRRK2 binds the Cdc42/Rac‐interactive binding (CRIB) domain of PAK6. Functionally, LRRK2 is required for PAK6 activation monitored by autophosphorylation of threonine 560 and PAK6‐dependent neurite outgrowth in mouse brain. Related to disease, we found that PAK6 is hyperactivated in G2019S and idiopathic PD (iPD) post‐mortem brains compared to healthy controls, highlighting PAK6 as a novel pharmacological target in PD.

## Materials and methods

### Animals

C57BL/6 LRRK2 wild‐type and knock‐out mice were provided by Dr. Heather Melrose and Jackson Laboratory [B6.129X1(FVB)‐*Lrrk2* ^*tm1.1Cai*^/J]. Housing and handling of mice were done in compliance with national guidelines. All animal procedures were approved by the Ethical Committee of the University of Padova and the Italian Ministry of Health (license 46/2012), and by the Institutional Care and Use Committee of KU Leuven.

### Plasmids

Eukaryotic expression constructs of 3xFlag tagged LRRK2 (wild type, K1906M and G2019S), and LRRK2 domains (in pCHMWS‐3xFlag vectors) were generated as described previously (Lobbestael *et al*. 2010; Daniels *et al*. 2011; Civiero *et al*. 2012). The pDONR223‐PAK6 (plasmid 23833, Johannessen *et al*. 2010) was obtained from Addgene (Cambridge, MA, USA). PAK cDNA sequences in pDONR223 were cloned by LR Clonase‐mediated gateway recombination (Life Technologies, Grand Island, NY, USA) into the destination vector pCMV‐Tag3B‐2xMyc modified with a gateway cassette as previously described (Greggio *et al*. 2007). Full‐length PAK6 was amplified from pDONR223‐PAK6 with the following primers: forward 5′‐GGTGCGGCCGCGATGTTCCGCAAGAAAAAG‐3′ and reverse 5′‐GGTTCTAGATCAGCAGGTGGAGGTCTG‐3′ that introduced NotI and XbaI restriction sites at the 5′ and 3′ ends of the PCR fragment, respectively. The PCR fragment was purified, cleaved with NotI/XbaI and cloned into 3xFlag‐cytomegalovirus (CMV) vector (Sigma, St Louis, MO, USA). Mutant variants were generated using the Quick‐Change II site‐directed mutagenesis kit (Stratagene, La Jolla, CA, USA).

For lentiviral vector (LV) construction, cDNA sequences encoding PAK6 wild type, K436M and S531N were amplified by PCR using oligonucleotides that introduced XbaI/XbaI restriction sites (forward 5′‐GGTTCTAGAATGTTCCGCAAGAAAAAG‐3′; reverse 5′‐ GGTTCTAGATCAGCAGGTGGAGGTCTG‐3′) and subcloned into the lentiviral transfer backbone pCHMWS‐3xFlag‐MCS‐ires‐eGFP (Ibrahimi *et al*. 2009).

To produce stable cell lines over‐expressing PAK6 wild type, the cDNA sequence was amplified by PCR using oligonucleotides that introduce ClaI/XbaI restriction sites (forward 5′‐GGTATCGATACCATGTTCCGCAAGAAAAAG‐3′; reverse 5′‐ GGTTCTAGATCAGCAGGTGGAGGTCTG‐3′ and subcloned into the lentiviral transfer plasmid pCHMWS‐MCS‐ires‐hygro.

To subclone PAK6 into the adeno‐associated viral transfer plasmid pAAV‐TF‐CMV‐GFP‐MCS, cDNA sequences encoding 3xFlag‐PAK6 wild type, K436M and S531N were amplified using oligonucleotides that introduce AgeI/XbaI restriction sites (forward 5′‐AAAAAAACCGGTGCCACCATGGACTACAAAGACCATGA‐3′; reverse 5′‐AAAAAATCTAGATCAGCAGGTGGAGG‐3′). The GFP sequence was excised from pAAV‐TF‐CMV‐GFP (Taymans *et al*. 2007) and the 3xFlag‐PAK6 cDNA fragments were inserted.

For glutatione S‐transferase (GST) pull‐down assay, the nucleotide sequence encoding CRIB was cloned into a pGEX‐4T bacterial vector. Two sets of primers with complementary overhangs encoding the CRIB sequence and containing EcoRI/XhoI restriction sites (forward A 5′‐ AATTCACATGGAGATCTCAGCGCCACAGAACTTCCAGCACCGTGTCCACACCTCCT‐3′; reverse A 5′‐GGTCGAAGGAGGTGTGGACACGGTGCTGGAAGTTCTGTGGCGCTGAGATCTCCATGTG‐3′; forward B 5′‐ TCGACCCCAAAGAAGGCAAGTTTGTGGGCCTCCCCCCACAATGGCAGAACATCCTGGACTGAC‐3′; reverse B 5′‐ TCGAGTCAGTCCAGGATGTTCTGCCATTGTGGGGGGAGGCCCACAAACTTGCCTTCTTTGG‐3′) were annealed, phosphorylated, and subsequently cloned. All plasmids were verified by restriction analysis and DNA sequencing.

### Cell cultures and transfection

HEK293T cells were purchased from Life technologies and cultured in Dulbecco's modified Eagle's medium supplemented with 10% fetal bovine serum. Cell lines, were maintained at 37°C and in a 5% CO_2_ controlled atmosphere. HEK293T were transfected with plasmid DNA using polyethylenimine‎ (Polysciences, Warrington, PA, USA) according to the manufacturer's recommendations.

### Viral vector production and transduction

All experiments involving viral vectors were carried out under biosafety level 2 conditions. Housing and handling of mice were done in compliance with national guidelines; all animal procedures were approved by the Institutional Care and Use Committee of the KU Leuven.

LV and recombinant adeno‐associated viral vectors (rAAV2/7) encoding mCherry‐GFP, human 3xFlag‐PAK6 wild type, K436M and S531N under control of the CMV promoter were produced by the Leuven Viral Vector Core as described previously (Lobbestael *et al*. 2010; Van der Perren *et al*. 2011). For transduction of mouse striata, eight‐week‐old male C57bL/*6* mice were used for rAAV‐3xFlag‐PAK6 injections. Animals were anaesthetized and placed in a stereotactic head frame. After making a midline incision of the scalp, a burr hole was drilled in the appropriate location at one or both sites of the skull using Bregma as reference. The following coordinates were used: anteroposterior 0.5 mm; lateral 2.0 mm; dorsoventral 3.0 mm. Two microliters of rAAV vectors (titers ranging from 1.5 to 3.8 × 10^12^ genome copies/mL) were injected unilaterally in mouse striatum at a rate of 0.25 μL/min with a 30‐gauge needle on a 10‐μL Hamilton syringe. After injection, the needle was left in place for additional 5 min before being slowly withdrawn from the brain. Two weeks later, animals were deeply anesthetized using an overdose of pentobarbital. For immunohistochemistry, animals were transcardially perfused with saline solution followed by ice‐cold 4% paraformaldehyde in phosphate‐buffered saline. The brain was removed from the skull and post‐fixed overnight in 4% paraformaldehyde‐phosphate‐buffered saline at 4°C. Sections (50 μm) were stained using rabbit anti‐flag antibody (Sigma) as previously described (Lobbestael *et al*. 2010). The mean percentage of the transduced striatal area is calculated by measuring the transduced striatal area/total striatal area every five sections of the mouse striatum. Alternatively, the striata were dissected, homogenized, and subjected to western blot analysis, as described in (Taymans *et al*. 2006).

### Antibodies

For immunoblotting analysis the following antibodies were used: rabbit LRRK2 MJFF2 (Cat# 3514‐1, RRID:AB_10643781, 1 : 100; Epitomics), rabbit LRRK2 phospho‐S935 (Cat# 5099‐1, RRID:AB_ 11132319, 1 : 100; Epitomics, Cambridge, UK), rabbit LRRK2 phospho‐T2483 (Cat#156577, 1 : 2000; Abcam, Cambridge, UK), rabbit LRRK2 phospho‐T1491 (Cat#140106, 1 : 2000; Abcam,Cambridge, UK), mouse Flag M2 (Cat# F1804, 1 : 10000; Sigma), mouse c‐Myc 9E10 (Cat# 11667149001, RRID:AB_ 390912, 1 : 5000; Roche Molecular Biochemicals, Indianapolis, IN, USA), rabbit PAK6 (Cat# HPA031124, RRID:AB_10601044, 1 : 2000, Prestige^®^; Sigma), mouse β‐tubulin (Cat# T8328, RRID:AB_1844090, 1 : 5000; Sigma), rabbit phospho‐PAK4‐5‐6 (Cat# SAB4504052, 1 : 2000; Sigma), rabbit phospho‐LIM domain kinase 1 (LIMK1) (Cat#3841, 1 : 1000; Cell Signaling Technology, Beverly, MA, USA), mouse LIMK1 (Cat#117623, 1 : 1000; Abcam).

For immunoprecipitation, the following antibodies were used: rabbit LRRK2 UDD3 (Cat# 5097‐1, 1 μg/mg total proteins; Epitomics), mouse c‐Myc (9E10, Cat# 11667149001, RRID:AB_390912, 0.8 μg/mg total proteins; Roche), mouse Flag M2 (Cat# F1804, 1 μg/mg total proteins; Sigma).

### Immunohistochemistry and confocal imaging

For the *in vivo* experiments on striatal neurons of normal and LRRK2 knock‐out mice expressing 3xFlag‐PAK6 variants (or mCherry control) and labeled for eGFP to study morphology, sections were analyzed by immunohistochemistry to detect eGFP and PAK6 or mCherry expressing neurons. Sections were incubated with rabbit anti‐eGFP and mouse anti‐flag antibody as described in Lobbestael *et al*. (Lobbestael *et al*. 2010). Labels were revealed with fluorescent secondary antibodies (anti‐rabbit‐alexa‐488 and anti‐mouse‐alexa‐555) and visualized by confocal microscopy. First eGFP‐labeled striatal neurons were confirmed to co‐express PAK6 variants or the mCherry control. Next, z‐stacks were taken of the confirmed neurons. 2D projections derived from these z‐stacks were submitted to neurite complexity analysis using the NeuronJ plugin in ImageJ (Meijering *et al*. 2004).

### Co‐immunoprecipitation and western blotting

Cells were harvested at 48 h post transfection and lysed in buffer containing 50 mM Tris pH 7.5, 1% Triton X‐100, 1 mM sodium orthovanadate, 5 mM sodium pyrophosphate, 50 mM sodium fluoride, 0.27 M sucrose, 1 mM EDTA). Lysates were incubated with primary antibody overnight then with Protein‐G Sepharose for 1 h or with primary antibody directly conjugated to agarose beads. Immunocomplexes were washed three times with lysis buffer supplemented with 0.25 M NaCl. Immunoprecipitates were resuspended in sample buffer.

Between 10 and 20 μg of protein samples were resolved on 4‐20% Tris‐glycine polyacrylamide gels (Bio‐Rad Laboratories, Hercules, CA, USA) in sodium dodecyl sulfate/Tris‐glycine running buffer or on NuPAGE^®^ 3–8% Tris‐acetate Gel (Life Technologies). Precision Plus molecular weight markers (Bio‐Rad) were used for size estimation. Resolved proteins were transferred to polyvinylidene difluoride (PVDF) membranes in transfer buffer containing 10% methanol. The PVDF sheets were blocked in Tris‐buffered saline plus 0.1% Triton (TBS‐T) plus 5% non‐fat dry milk for 1 h at 4°C and then incubated overnight at 4°C with anti‐Flag‐M2 antibody in TBS‐T plus 5% non‐fat dry milk. The PVDF membranes were washed in TBS‐T (3 × 10 min) at RT followed by incubation for 1 h at RT with horseradish peroxidase‐conjugated anti‐mouse IgG. Blots were then washed in TBS‐T (4 × 10 min) at RT and rinsed in TBS, and immunoreactive proteins were visualized using enhanced chemiluminescence plus (GE Healthcare, Little Chalfont, England). Densitometric analysis was carried out using Image J software (Schneider *et al*. 2012).

### Pull‐down assay

GST‐tagged proteins were expressed and purified from BL21 bacterial cells (as described in Greggio *et al*. 2009); 3xFlag‐tagged proteins were expressed in HEK293T cell lines as previously described (Civiero *et al*. 2012). Purified proteins bound to the resin were incubated for 2 h with cell lysates over‐expressing the prey protein. For the following procedure see co‐immunoprecipitation and western blotting section.

### *In vitro* kinase reactions

Kinase assays were carried out as previously described (Civiero *et al*. 2012; Jebelli *et al*. 2012). Protein concentrations used are indicated in figure legends.

### Post‐mortem human tissues analysis

Post‐mortem human tissue samples were obtained from Queen Square Brain Bank (London, UK). Sample demographics are listed in Table 1. The 5% sodium dodecyl sulfate fractions from the basal ganglia from three G2019S LRRK2 mutation cases, four matched iPD cases and four control cases were prepared according to the methods described in Mamais *et al*. (Mamais *et al*. 2013). 40 μg of proteins was loaded onto 12% Tris‐Glycine gels (Bio‐Rad) and transferred onto PVDF membrane. The membranes were probed with primary antibodies at 1 : 1000 dilution (phospho‐S602/560 antibody Sigma Cat no SAB4504722; PAK‐6 Sigma Cat no HPA031124). Immunohistochemistry with PAK phospho‐S602/560 antibody was performed on formalin fixed wax‐embedded slides. Briefly, sections 8‐μm‐thick were dewaxed in xylene, blocked for endogenous peroxidase with H_2_O_2_ (0.3%) containing methanol followed by pressure‐cooking in citrate buffer pH 7.0 for 10 min to reveal antigenic sites. The sections were then blocked in 10% non‐fat milk for an hour at 23°C followed by incubation in primary antibody at 1 : 100 dilution o/n at 4°C. Following washes, sections were treated with anti‐rabbit biotynilated secondary antibody (1 : 200, 30 min; Dako, Carpinteria, CA, USA) followed by treatment with ABC reagent (Vector Laboratories, Burlingame, CA, USA) for 30 min and visualizing with H_2_O_2‐_activated diaminobenzidine as chromogen. Sections are counter‐stained lightly with Mayer's hematoxylin, taken through graded ethanols and xylene and mounted with coverslips with DPX (VWR, International PBI, Milano, Italy) mounting medium.

**Table 1:** Sample demographics of the human cases used in this study

Case	Sex M/F	Age (years)	PM Delay (h)	pH of tissue	WB/IH
G2019S1	F	80	44.4	6	WB &IH
G2019S2	F	81	15	6.53	WB
G2019S3	F	84	32.2	5.79	WB&IH
G2019S4	F	72	24.55	6.2	WB&IH
iPD1	M	70	61.2	6.29	WB&IH
iPD2	F	87	47.45	6.62	WB&IH
iPD3	M	75	48	6.0	WB
iPD4	F	88	11.3	6.38	WB&IH
Control 1	F	85	37	6.4	WB&IH
Control 2	F	91	98.5	6.26	WB&IH
Control 3	M	87	36	6.1	WB&IH
Control 4	F	68	41.5	5.98	WB
AD	M	82	38	N/A	IH

iPD, idiopathic PD; N/A, not available.

A four‐tired grading system was used to provide a semi‐quantitative assessment of P‐PAK immunoreactivity (IR) in basal ganglia. Assessment was by consensus between two observers. Score 0 = no P‐PAK IR, score + = weak P‐PAK IR, score ++ = moderate P‐PAK IR, score +++ = strong P‐PAK IR.

### Statistical analysis

All quantitative data are expressed as mean ± SD (standard error) or SEM (standard error of the mean) and represent at least three independent sets of experiments. Significance of differences between two groups was assessed by unpaired *t*‐test or by one‐way anova with Tukey's *post hoc* test and two‐way anova with Tukey's HSD *post hoc* test when more than two groups were compared. Significance level was set at *p* < 0.05.

## Results

### LRRK2 interacts with PAK6

**Figure 1:**
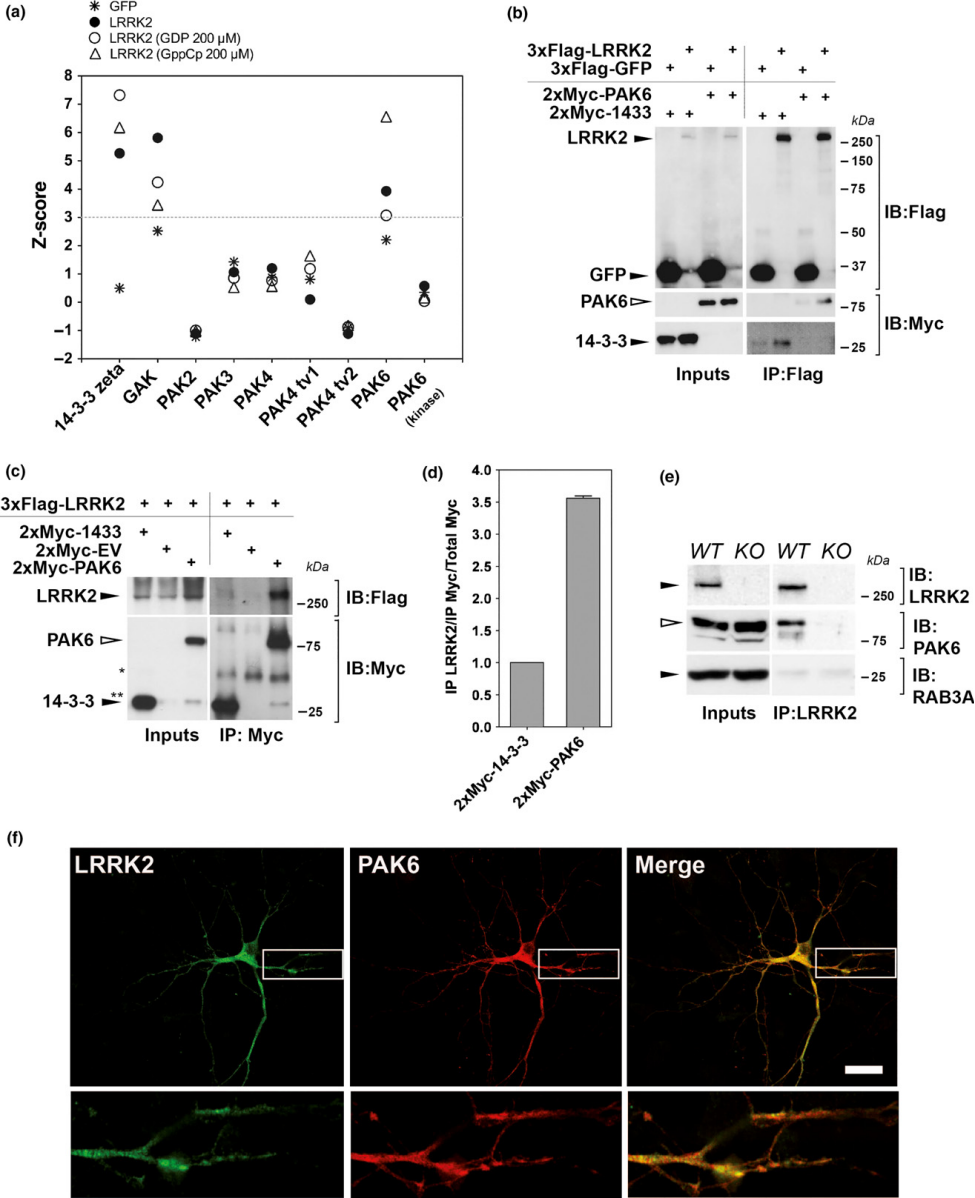
PAK6 interacts with LRRK2. (a) Plot of Z‐score (*y*‐axis) for selected bait proteins (*x*‐axis; 14‐3‐3 zeta and cyclin G‐associated kinase (GAK) are positive controls) for arrays probed with GFP (stars), LRRK2 alone (filled black dots), LRRK2 in the presence of GDP (empty black dots) and LRRK2 in the presence of Guanosine‐5′‐[(β,γ)‐methyleno]triphosphate (GppCp) (empty black triangles). The dotted horizontal line indicates Z = 3 used as a cutoff for identifying candidate hits. Note that, outside of the positive control proteins, only full‐length PAK6 is above the threshold line. (b) PAK6 co‐immunoprecipitates with LRRK2 *in vitro*. Cell lysates from HEK293T cells co‐transfected with Flag‐LRRK2 and Myc‐PAK6 or Myc‐14‐3‐3 were subjected to co‐immunoprecipitation with anti‐Flag, followed by anti‐Myc and anti‐Flag immunoblotting. As negative control, cell lysates co‐transfected with Flag‐GFP and Myc‐PAK6 or Myc‐14‐3‐3 were subjected to the same protocol. (c) Cell lysates from HEK293T cells co‐transfected with Flag‐LRRK2 and Myc‐PAK6 or Myc‐1433 were subjected to co‐immunoprecipitation with anti‐myc, followed by anti‐Myc and anti‐Flag immunoblotting. As negative control, cell lysates co‐transfected with Flag‐LRRK2 and empty vector (EV) were subjected to the same protocol. (d) Quantification of LRRK2 binding to PAK6 and 14‐3‐3. Data are representative of three independent experiments and bars represent the mean ± SEM relative to 14‐3‐3. (e) PAK6 interacts with LRRK2 *in vivo*. Endogenous LRRK2 was immunoprecipitated from wild‐type and LRRK2 knock‐out brain lysates as control using anti‐LRRK2 antibody. Samples were analyzed by immunoblotting using anti‐PAK6 and anti‐LRRK2. (f) PAK6 and LRRK2 co‐localize in neurons. Primary cortical neurons were transfected with Flag‐LRRK2 and Myc‐PAK6 and then subjected to immunocytochemistry techniques using anti‐Flag (green) and anti‐PAK6 (red) antibodies. * and ** indicate antibody chains

In a previous study, we reported high confidence interactors of LRRK2 identified by probing protoarrays with full‐length recombinant LRRK2 protein (Beilina *et al*. 2014; Reyniers *et al*. 2014). We repeated this experiment with purified full‐length LRRK2 alone or with additional GDP and non‐hydrolysable GTP (Guanosine‐5′‐[(β,γ)‐methyleno]triphosphate, GppCp) and used a Z‐score of 3 (i.e., 3 standard deviations from background) as a cutoff for candidate interactions. Together with known interactors including 14‐3‐3 proteins (Dzamko *et al*. 2010; Nichols *et al*. 2010) and cyclin G‐associated kinase (Beilina *et al*. 2014), the protoarray experiment identified p21‐activated kinase 6 (PAK6) as a potential LRRK2 interactor with higher interaction in the presence of GppCp (Z scores: apo‐LRRK2 3.93, GDP‐LRRK2 3.07, and GppCp‐LRRK2 6.56; Fig. 1a). Of the members of the PAK family (PAK2, PAK3, and two PAK4 transcript variants) spotted on the arrays, only PAK6 showed Z scores above the threshold of 3. PAK6 kinase domain alone also spotted on the array did not give significant signal (Fig. 1a). These results suggest that full‐length PAK6 is required to observe interaction with LRRK2.

PAK6 belongs to group II p21‐activated kinases (PAKs), a family of proteins involved in cell remodeling pathways *via* regulation of actin cytoskeleton dynamics (Szczepanowska 2009), processes where also LRRK2 has been implicated (Meixner *et al*. 2011; Habig *et al*. 2013; Caesar *et al*. 2015). We therefore pursued the hypothesis of PAK6 as a potential interactor with, and mediator of the biological effects of, LRRK2. We first performed co‐immunoprecipitation to confirm the interaction. In HEK293T cells, 2xMyc‐PAK6 was co‐immunoprecipitated with 3xFlag‐LRRK2 (Fig. 1b) and 3xFlag‐LRRK2 could be reciprocally co‐immunoprecipitated by 2xMyc‐PAK6 (Fig. 1c). Under these conditions, we recovered ~ 3.5‐fold more LRRK2 protein bound to 2xMyc‐PAK6 than to 2xMyc‐14‐3‐3 zeta (Fig. 1d). We subsequently tested the interaction between the two endogenous kinases in mouse brain. LRRK2 immunoprecipitated from wild‐type mouse brain efficiently co‐purifies PAK6, whereas no PAK6 is detected in knock‐out lysates incubated with anti‐LRRK2 antibodies (Fig. 1e). As negative control, we did not observe interaction with Rab3A. In addition, ectopic expression of LRRK2 and PAK6 in primary cortical neurons results in co‐localization of the two kinases in the soma and dendrites with both diffuse and spotted distribution (Fig. 1f).

**Figure 2:**
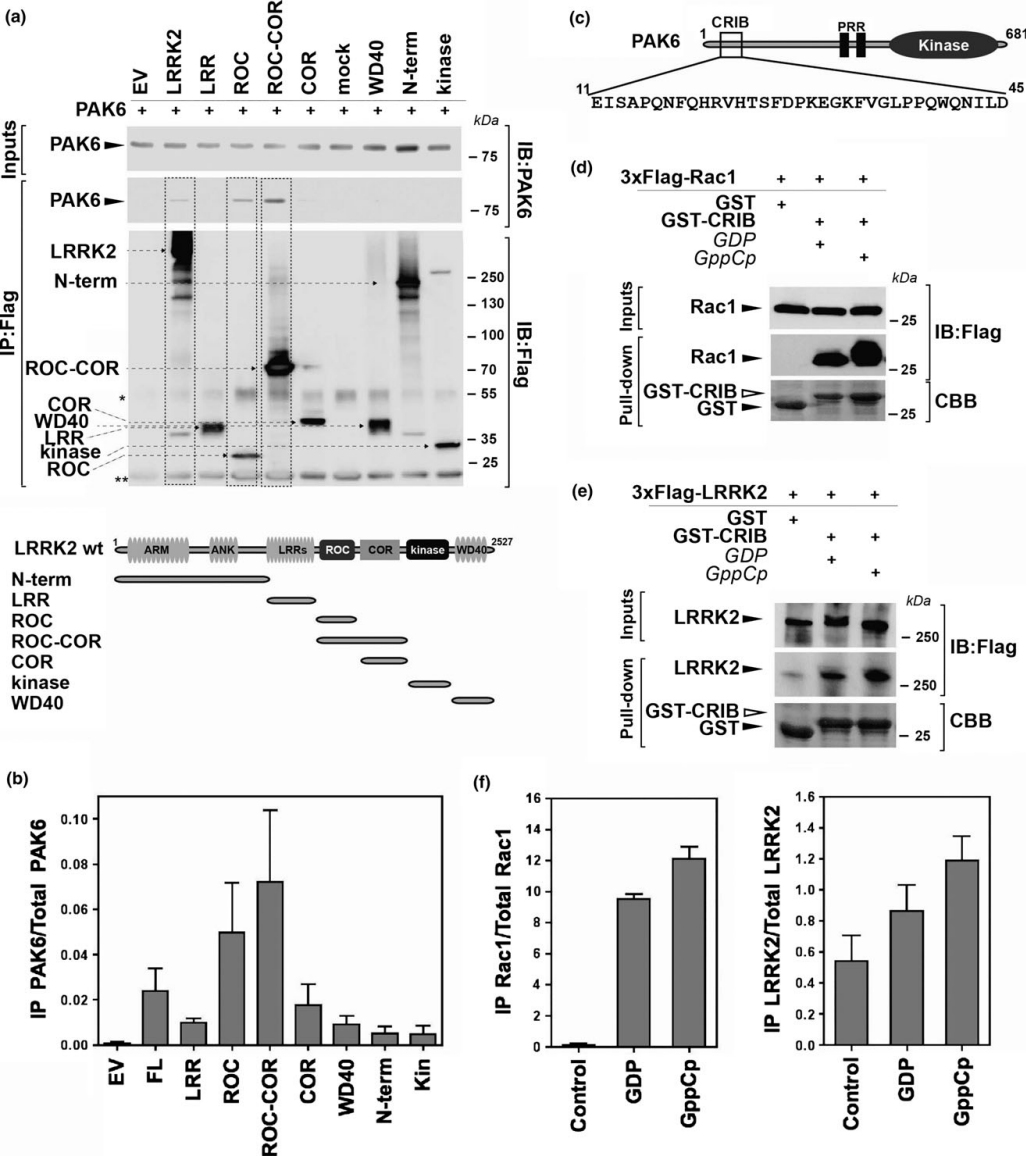
The Cdc42/Rac‐interactive binding (CRIB) domain of PAK6 interacts with the ras of complex (ROC) domain of LRRK2. (a) The ROC domain of LRRK2 is responsible for LRRK2‐PAK6 interaction. Cell lysates from stable HEK293T cells over‐expressing PAK6 and transfected with an empty vector (EV), Flag‐tagged full‐length LRRK2 or LRRK2 fragments were subjected to co‐immunoprecipitation using anti‐Flag antibody, followed by anti‐PAK6 and anti‐Flag immunoblotting. A schematic representation of the different LRRK2 fragments used is shown below immunoblots. Blots are representative of three independent experiments. * and ** indicate antibody heavy and light chains, respectively. (b) Quantification of PAK6 binding to LRRK2 domains. Data are representative of three independent experiments and bars represent the mean ± SEM. (c) Schematic of PAK6 domains and amino acid sequence of CRIB domain. (d) GST‐CRIB of PAK6 detects active Rac1. GST‐CRIB and GST alone purified from bacterial sources and bound to glutathione‐sepharose resin were incubated with cell lysates from HEK293T cells over‐expressing 3xFlag‐Rac1 in the presence of GDP or non‐hydrolyzable GTP. Samples were subjected to immunoblotting using anti‐Flag or stained with Coomassie. (e) PAK6 interacts with LRRK2 *via* CRIB. GST‐CRIB and GST alone purified from bacterial sources and bound to glutathione‐sepharose resin were incubated with cell lysates from HEK293T cells transfected with 3xFlag‐LRRK2 in the presence of GDP or non‐hydrolyzable GTP. Samples were subjected to immunoblotting using anti‐Flag or stained with Coomassie. (f) Quantification of Rac1 and LRRK2 binding to the CRIB domain. Data are representative of three independent experiments and bars represent the mean ± SEM.

We then dissected the interaction down to domain level. PAK6 interacted with constructs containing the ROC and ROC‐COR, but not COR domain, of LRRK2 (Fig. 2a–b). As we had observed that the interaction is modulated by guanine nucleotides in protoarrays and involves the ROC domain of LRRK2, we hypothesized that PAK6 interacts with LRRK2 *via* its CRIB domain, a conserved sequence near the N‐terminus (Fig. 2c) involved in the binding of small GTPase such as Cdc42 and Rac1 (Thompson *et al*. 1998) (PDB‐ID: 2ODB). To test this, we performed a pull‐down assay generally used to isolate active small GTPases such as Rac1. As a control, we first incubated GST‐tagged CRIB bound to glutathione‐sepharose beads with cell lysates from HEK293T cells expressing 3xFlag‐Rac1 in the presence of GDP or GppCp. As expected, Rac1 strongly binds PAK6 GST‐CRIB when activated with non‐hydrolysable GTP (Fig. 2d–f). We then performed the experiment using lysates from HEK293T transfected with 3xFlag‐LRRK2. GST‐CRIB pulls‐down LRRK2 protein and displays increased interaction for the kinase in the presence of non‐hydrolyzable nucleotides (Fig. 2e–f).

These data indicate that LRRK2 and PAK6 interact through ROC and CRIB domain, respectively, in a guanine nucleotide‐dependent fashion.

### PAK6 induces neurite outgrowth in a manner dependent on its kinase activity and LRRK2

We next asked whether LRRK2‐PAK6 interaction might have functional consequences in neurons. PAKs play a central role in dendrite development, contributing to branching and spine formation by modulating actin and microtubule dynamics in a kinase‐dependent manner (Eswaran *et al*. 2008). Double PAK5/6 knock‐out mice display a decreased number of neuronal processes, locomotor changes, and memory deficits (Nekrasova *et al*. 2008). Similarly, LRRK2 has an established role in regulating neurite outgrowth (MacLeod *et al*. 2006; Dachsel *et al*. 2010; Sepulveda *et al*. 2013). To investigate a putative role of LRRK2 and PAK6 in regulating neuronal morphology *in vivo*, we measured the effect of PAK6 expression on neurite length in LRRK2 wild‐type versus knock‐out mouse striatum.

**Figure 3:**
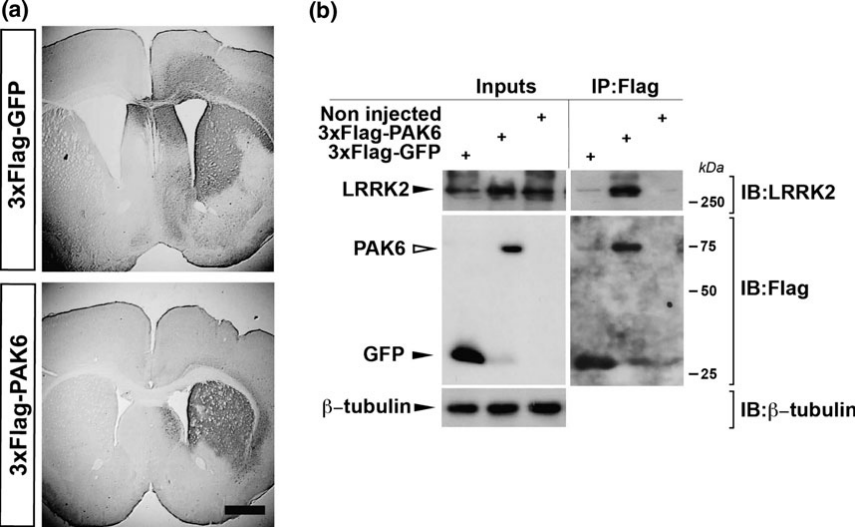
PAK6 binds endogenous LRRK2 *in vivo*. (a) Striatal sections (50 μm) of paraformaldehyde‐perfused brains sterotaxically injected with recombinant adeno‐associated viral (rAAV) encoding 3xFlag‐PAK6 and 3xFlag‐GFP and incubated with anti‐flag antibody followed by 3, 3′‐diaminobenzidine‐peroxidase staining. (b) Lysates from mouse striatum injected with rAAVs encoding 3xFlag‐PAK6 and 3xFlag‐GFP were subjected to co‐immunoprecipitation using anti‐Flag antibody, followed by anti‐Flag and anti‐LRRK2 (MJFF2) immunoblotting. Scale bar 1 mm.

First, the interaction between over‐expressed PAK6 and endogenous LRRK2 was assessed in our paradigm. rAAV2/7 vectors expressing 3xFlag‐PAK6 wild type or 3xFlag‐GFP were unilaterally injected in the striatum of 3‐month‐old C57bL/6 mice and 15 days post injection, transgene expression and spreading were confirmed by immunohistochemistry and western blotting analysis (Fig. 3a). PAK6 immunoprecipitated from dissected striata interacted strongly with endogenous LRRK2, whereas Flag immunoprecipitations of GFP‐expressing striatum or non‐injected striatum failed to co‐immunoprecipitate LRRK2 (Fig. 3b).

**Figure 4:**
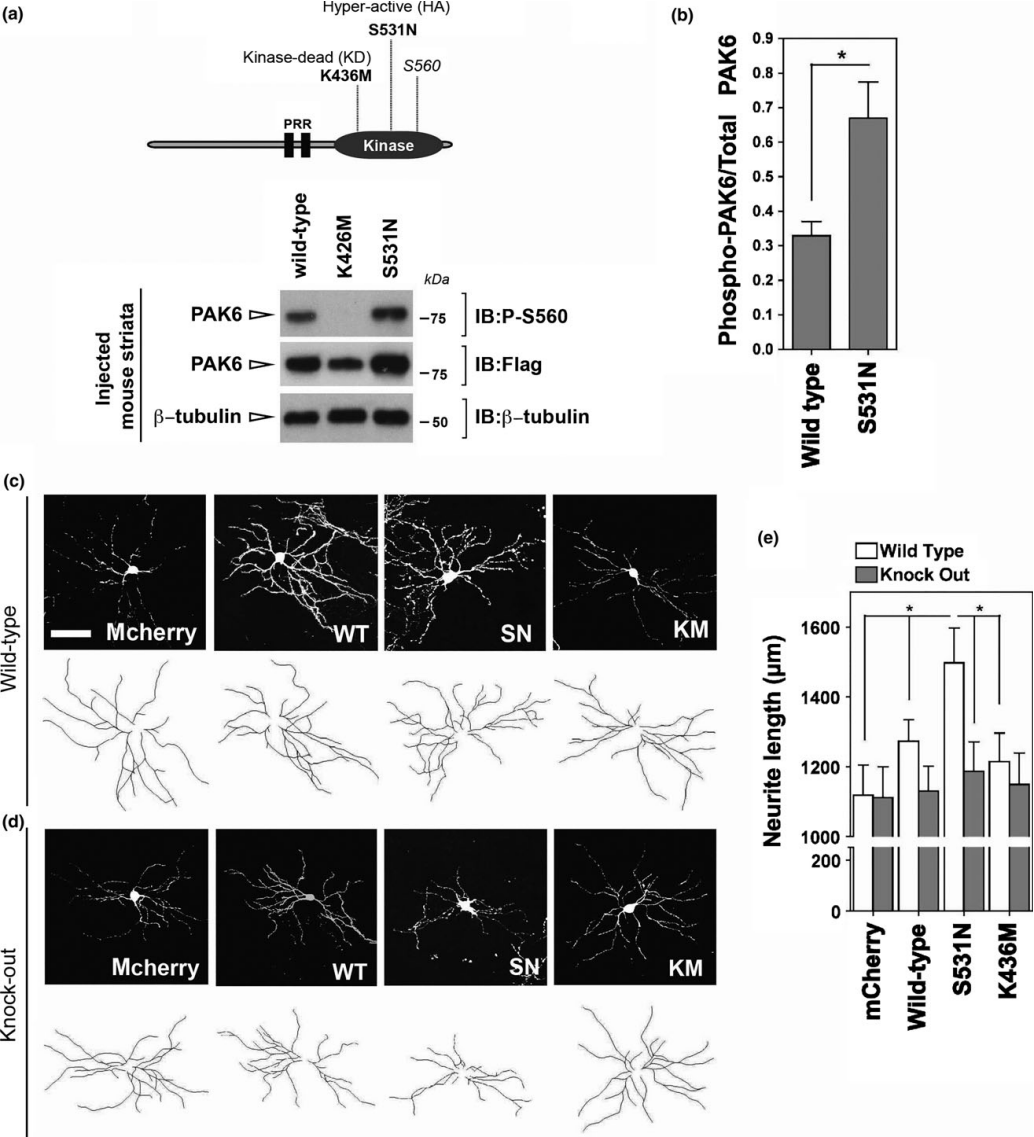
PAK6 and LRRK2 cooperate to control neurite growth *in vivo*. (a) Schematic of PAK6 functional mutations. Auto‐phosphorylation of PAK6 at S560 is monitored by western blotting of striata injected with PAK6 wild type, K436M and S531N probed with anti phospho‐S560 and anti‐PAK6 antibodies. (b) Quantification of (a) from *n* = 4 injected brains (bars represent the mean ± SEM, unpaired *t*‐test; *p <* *0.05). (c–d) Representative images of striatal neurons co‐transduced with recombinant adeno‐associated virals encoding PAK6 wild type, S531N, K436M or mCherry as control together with low titer LV‐eGFP to label individual neurons. Scale bar represents 50 μm. (e) Quantification of neurite length by two‐way anova with Tukey's HSD *post hoc* test for all variants (*p <* *0.05). Data were collected from six injected striata per condition. Twelve transduced neurons per condition were analyzed (bars represent the mean ± SEM).

To explore the impact of PAK6 kinase activity in regulating neuronal branching, we then generated PAK6 kinase dead (K436M) and hyper‐active (S531N) mutants (Fig. 4a) (Schrantz *et al*. 2004). As readout of PAK6 kinase activity in our experimental model, we monitored the autophosphorylation of PAK6 at S560, a site conserved among PAK4/5/6 and analogous to T423 of PAK1, known to play a pivotal role in regulating the activity and function of PAK6 (Qu *et al*. 2001). As expected, PAK6 K436M is devoid of autophosphorylation activity, whereas PAK6 S531N is ~ 2‐fold more active than its wild‐type counterpart (Fig. 4a–b) (*p < 0.05 PAK6 wild type vs. S531N, unpaired *t*‐test).

Then, rAAV encoding mCherry control, 3xFlag‐PAK6 wild type, 3xFlag‐K436M and 3xFlag‐S531N were injected in mouse striatum at titers to obtain broad expression and co‐injected with LV‐eGFP at a low titer to label isolated neurons for the subsequent morphological analysis (Figure S1a). The efficiency of the rAAV vectors in transducing PAK6 variants at comparable levels in mouse striata was tested by 3, 3′‐diaminobenzidine staining (Figure S1b). Six striata were injected per condition and neuronal morphology was analyzed on z‐stack projections obtained by confocal microscopy. In our system, neurite complexity is not changed by the ablation of *LRRK2* gene in adult striatum (Fig. 4c–d), which contrasts to what is observed in primary cultures (Dachsel *et al*. 2010). This suggests that the enhanced neurite outgrowth phenotype observed in knock‐out primary neurons is presumably related to development more than maintenance of neurites in the adult. Instead, we observed that expression of PAK6 wild type results in a modest increase in neurite length compared to control, whereas expression of the hyper‐active PAK6 S531N caused a significant increase in wild‐type mice (Fig. 4c–e; *p* < 0.05, two‐way anova with Tukey's HSD *post hoc* test). Strikingly, PAK6 S531N is no longer able to stimulate neurite outgrowth in LRRK2 knock‐out neurons (Fig. 4d–e, two‐way anova with Tukey's HSD *post hoc* test). As control, we do not observe any morphological changes between wild type and knock‐out striatal neurons transduced with PAK6 K436M.

Taken together, these results indicate that PAK6 kinase activity enhances neurite length and complexity through LRRK2.

### LRRK2 regulates PAK6 activation *in vivo*

**Figure 5:**
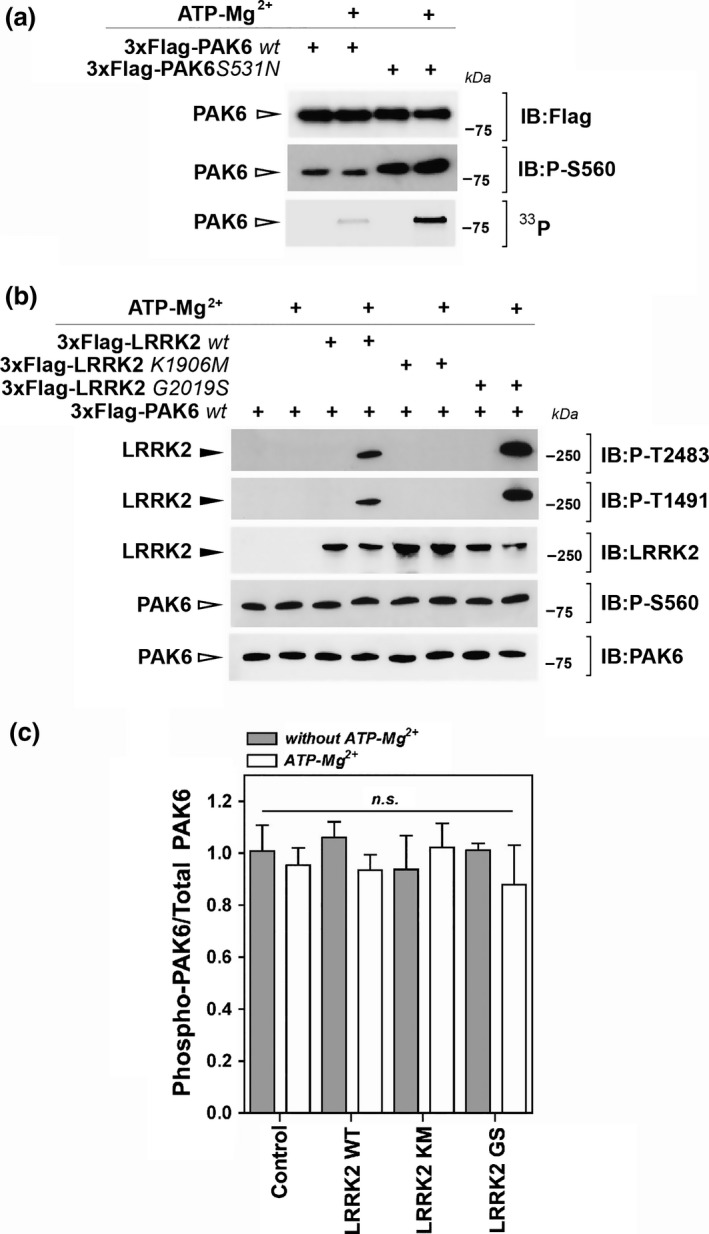
LRRK2 does not stimulate PAK6 auto‐phosphorylation *in vitro*. (a) PAK6 activation at S560 occurs in the cell. Recombinant 3xFlag‐PAK6 wild type and S536N were purified and subjected to kinase assays *in vitro* with or without the addition of ATP‐Mg^2+^. PAK6 activation and kinase activity were measured by monitoring S560 and the incorporation of ^33^P, respectively. (b) PAK6 activation at S560 is not regulated by LRRK2 *in vitro*. Recombinant 3xFlag‐PAK6 alone or together with 3xFlag‐LRRK2 wild type, K1906M and G2019S in a 1 : 3 ratio was subjected to kinase assays *in vitro* with or without the addition of ATP‐Mg^2+^. The incorporation of ^33^P was monitored by western blotting with anti phospho‐S560 PAK6 and anti phospho‐T2483/T1491 LRRK2 antibodies. (c) Quantification of phospho‐S560 by one‐way anova with Tukey's HSD *post hoc* test. Data were collected from three independent experiments (bars represent the mean ± SEM).

**Figure 6:**
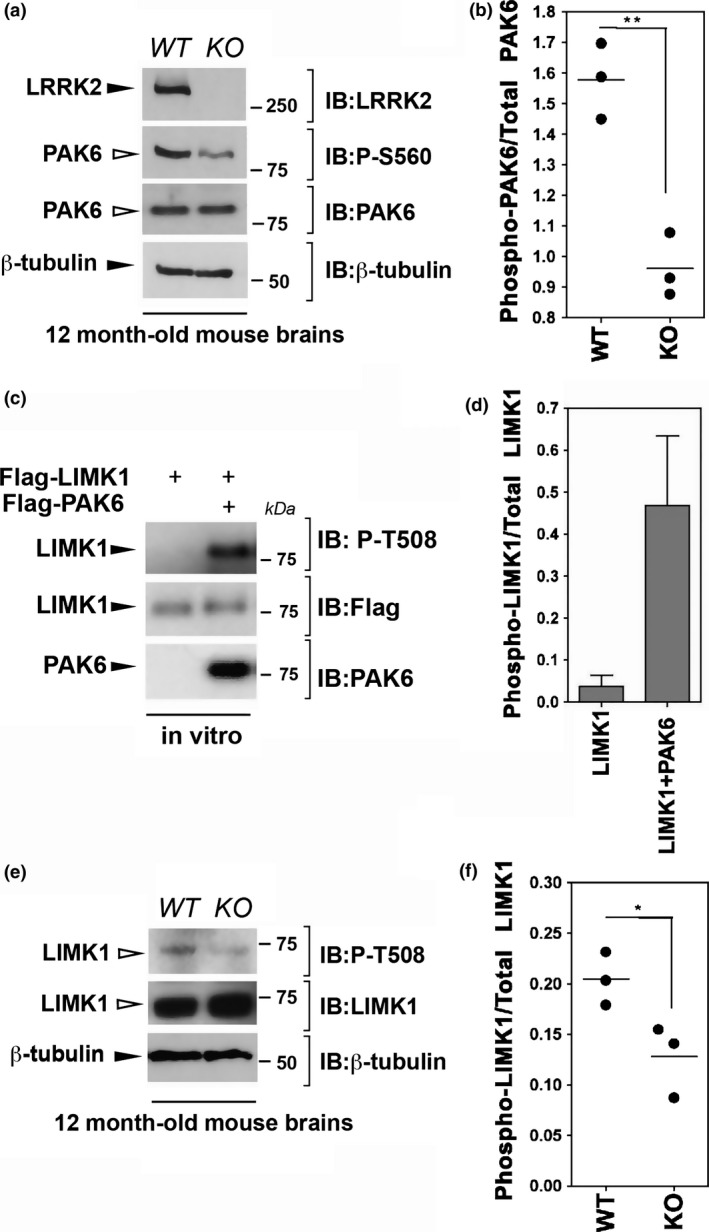
PAK6 and LIMK1 activation are impaired in LRRK2 knock‐out mouse brains. (a) PAK6 phosphorylation is decreased in LRRK2 knock‐out brains. Lysates from LRRK2 three wild‐type and three knock‐out mouse brains were subjected to immunoblot using anti PAK6 and anti phospho‐S560. (b) Quantification of phospho‐S560 by unpaired *t*‐test (**p < 0.01). Data were collected from three 12‐month old‐mouse brains (bars represent the mean ± SEM). (c) PAK6 phosphorylates LIMK1 at T508. Recombinant 3xFlag LIMK1 and PAK6 were subjected to kinase assays in a 5 : 1 ratio. The amount of phosphorylated LIMK1 was quantified by western blotting with anti phospho‐T508 LIMK1 antibody. (d) Quantification of phospho‐T508 relative to total LIMK1. Data were collected from three independent kinase assays (bars represent the mean ± SEM). (e) LIMK1 phos‐phorylation is decreased in LRRK2 knock‐out brains. Lysates from LRRK2 three wild‐type and three knock‐out mouse brains were subjected to immunoblot using anti‐LIMK1 and anti anti phospho‐T508 antibodies. (f) Quantification of phospho‐T508 by unpaired *t*‐test (*p < 0.05). Data were collected from three 12‐month old‐mouse brains (bars represent the mean ± SEM).

As the results presented so far suggest that PAK6 requires LRRK2 to exert its function, we next investigated whether LRRK2 can activate PAK6. To this aim, we first tested the ability of LRRK2 to stimulate PAK6 autophosphorylation *in vitro*. 3xFlag‐PAK6 wild type and S531N were purified and either subjected, or not, to *in vitro* kinase assays. While the S531N exhibits ~ 3‐fold higher phosphorylation at S560 compared to wild type, as expected, both proteins were unable to further autophosphorylate at this site *in vitro* (Fig. 5a). This suggests that additional cellular components are required to stimulate autophosphorylation of S560. We then asked whether LRRK2 is sufficient to trigger this phosphorylation. 3xFlag‐LRRK2 wild type, G2019S and K1906M were purified and incubated with 3xFlag‐PAK6 in the presence or absence of Mg^2+^ and ATP. Phosphorylation of T2483 and T1491 (two LRRK2 autophosphorylation sites) was monitored to confirm that the kinase reaction worked (Fig. 5b). Under this assay condition, we found that autophosphorylation of PAK6 at S560 was not stimulated by LRRK2 kinase activity (G2019S) or by LRRK2 itself (K1906M) (Fig. 5b–c, *p* > 0.05 for all groups, one‐way anova with Tukey's HSD *post hoc* test). Altogether, these results indicate that isolated LRRK2 is not able to activate isolated PAK6 *in vitro* and that a more complex cellular mechanism is likely required. To test this second hypothesis, we monitored the activation status of the kinase in brain lysates from LRRK2 wild type versus knock‐out mice. While we could not observe any difference in PAK6 autophosphorylation in 3‐month‐old brains (data not shown), a significant decrease in S560 phosphorylation was found in 12‐month‐old LRRK2 knock‐out compared to wild‐type mice (Fig. 6a–b, **p < 0.01, unpaired *t*‐test).

To further investigate if the observed LRRK2‐dependent PAK6 activation has an impact on the downstream components of the signaling pathway, we then compared the phosphorylation status of a PAK6 substrate, LIMK1, in brains from LRRK2 wild‐type versus knock‐out mice. LIMK1 is an established downstream effector of the PAK family (Radu *et al*. 2014), which plays a key role in the regulation of actin polymerization through downstream phosphorylation of the actin‐severing protein cofilin (Yang *et al*. 1998). First, we tested the ability of PAK6 to phosphorylate LIMK1 at T508 *in vitro*. Recombinant 3xFlag‐PAK6 wild type and 3XFlag‐LIMK1 were purified and subjected to *in vitro* kinase assay. As shown in Fig. 6c–d, PAK6 can efficiently phosphorylate LIMK1 at T508. We subsequently measured the phosphorylation levels of LIMK1 at T508 in brain lysates from LRRK2 wild‐type and knock‐out mice and observed a significant reduction of phospho‐T508 in knock‐out brains, similar to what was observed for PAK6 S560 (Fig. 6e–f, *p < 0.05, unpaired *t*‐test).

Overall, these results suggest that LRRK2 is part of a cellular complex required to activate the PAK6 pathway *in vivo*.

### PAK6 is aberrantly activated in post‐mortem tissues from PD brains

**Figure 7:**
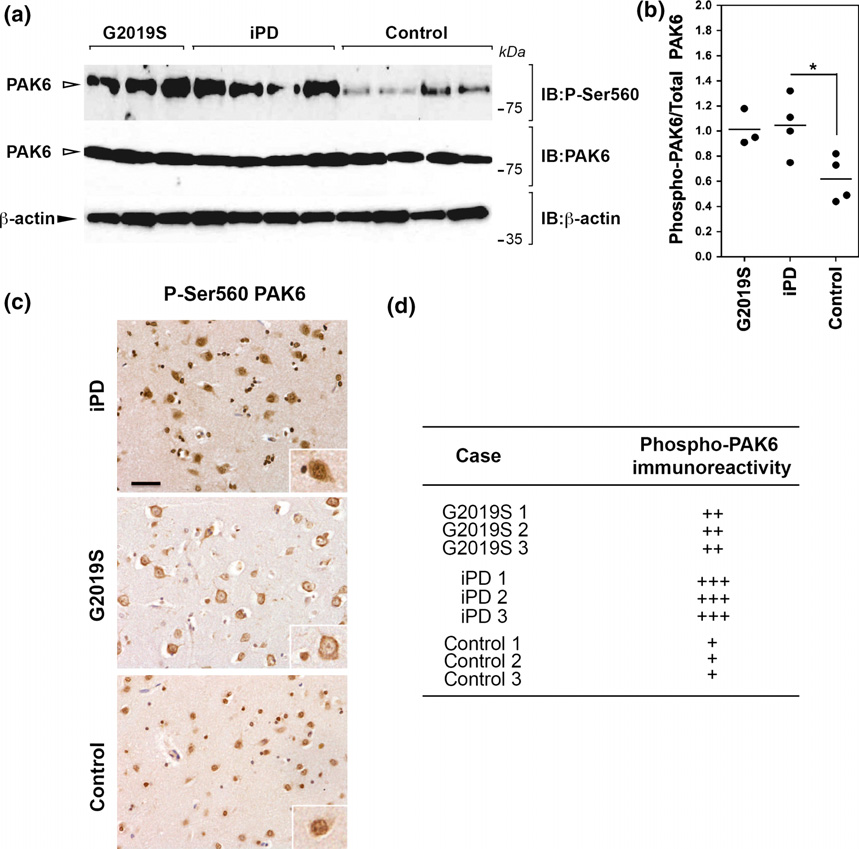
Phospho‐PAK6 levels are increased in mutant G2019S LRRK2 and idiopathic PD (iPD) brains. (a) Immunoblot of endogenous phospho‐S560 PAK6 levels in basal ganglia lysates from human G2019S LRRK2 Parkinson's disease (PD) patients, human iPD patients, and neurologic controls. (b) Quantification of phospho‐S560 PAK6 by one‐way anova with Tukey's post hoc test (*p < 0.05, iPD vs. control). (c) Representative images of phospho‐S560 PAK6 staining in basal ganglia from iPD, G2019S LRRK2 PD patients and a control case. Scale bar 10 μM. (d) Semi‐quantitative analysis of phospho‐S560 PAK6 immunoreactivity (IR) in G2019S, iPD and control cases. Phospho‐S560 IR was assessed according to a four‐tired scoring system: score 0 = no P‐PAK6 IR, score + = weak P‐PAK6 IR, score ++ = moderate P‐PAK6 IR, score +++ = strong P‐PAK6 IR.

To investigate if PAK6 is aberrantly activated in pathological conditions, we next measured PAK6 S560 phosphorylation in G2019S and iPD brains. Western blot analysis shows that phospho‐PAK6 is increased in basal ganglia from iPD (*n* = 4 cases) as well as mutant G2019S LRRK2 (*n* = 3 cases) PD patients of ~ 2‐fold compared to age‐matched healthy controls (*n* = 4 cases) (Fig. 7a–b). These results were further supported by immunohistochemistry of basal ganglia sections from iPD and G2019S LRRK2 patients versus controls (Fig. 7c–d).

Taken together, these data support a functional interplay between LRRK2 and PAK6 in the pathophysiology of human PD and suggest that LRRK2 may exert its toxicity through an aberrant regulation of PAK6 in PD.

## Discussion

Mutations in *LRRK2* are a common cause of PD; however, the physiological function of LRRK2 and the molecular mechanisms behind LRRK2‐linked PD are still poorly understood. One approach to gain insights into the function/dysfunction of a protein of interest is to elucidate its interactome. Here, we reveal the functional nature of the interaction between LRRK2 and PAK6, a novel LRRK2 partner identified using a protein array screening methodology. We demonstrated that LRRK2 and PAK6, interacting through their GTPase/ROC and CRIB domains, form a functional complex in mammalian brain, which impacts neurite outgrowth.

PAKs comprise a family of serine‐threonine kinases playing a central role in signal transduction. In contrast to class I PAKs (PAK1‐3) which are activated by Rho GTPase binding, class II PAKs (PAK4‐6) are re‐localized (not activated) by GTPases within specific signaling sites and locally activated by binding with SH3 domains to release pseudo‐substrate inhibition (Ha *et al*. 2012). One of the best‐characterized functions of these kinases is their role in actin cytoskeleton reorganization, such as formation of lamellipodia, filopodia, and membrane‐ruffles *via* the LIM kinase‐cofilin pathway (Edwards *et al*. 1999). PAK5 and PAK6 are highly expressed in the brain and, interestingly, PAK5/PAK6 double knock‐out mice display neurite shortening and learning and memory defects (Nekrasova *et al*. 2008).

In the nervous system, finely controlled neuronal connectivity is fundamental for maintenance of brain architecture and cognitive functions. Dynamic changes in actin cytoskeleton provide the mechanical force for neurite outgrowth, synapse formation and neuronal migration. Accordingly, defective cytoskeletal dynamics causes multiple neurodegenerative diseases (Heredia *et al*. 2006; Ma *et al*. 2012; Saal *et al*. 2015). LRRK2 has been robustly linked to actin dynamics: it impacts Erzin, Radixin, Moesin phosphorylation in neurons (Parisiadou *et al*. 2009) and binds F‐actin modulating its assembly *in vitro* (Meixner *et al*. 2011). Furthermore, phosphorylation of LRRK2 at S910/935 is required for binding to 14‐3‐3 proteins, and LRRK2 dephosphorylation results in protein re‐localization within defined intracellular sites including cytoskeletal‐associated structures (Dzamko *et al*. 2010; Nichols *et al*. 2010). To explore the hypothesis of an interplay between LRRK2 and PAK6 in a signaling network related to actin cytoskeleton dynamics**,** we searched for a functional phenotype *in vivo*. We found that over‐expression of PAK6 in brain striata increases neurite length in a kinase dependent manner. However, when LRRK2 is knocked‐out, PAK6 activity is no longer effective, supporting the notion that LRRK2 is required for PAK6‐dependent regulation of neurite morphogenesis.

Rho GTPase‐dependent neurite elongation and branching are essential mechanisms for the formation of functional networks connecting neurons through synapses, and its deregulation may contribute to neurodegeneration (Heredia *et al*. 2006; DeGeer and Lamarche‐Vane 2013; Saal *et al*. 2015). Interestingly, LRRK2 has been previously suggested to influence neurogenesis (Buchwald *et al*. 2001; Winner *et al*. 2011) as well as pre‐synaptic (Matta *et al*. 2012; Cirnaru *et al*. 2014) and post‐synaptic functions (Migheli *et al*. 2013; Beccano‐Kelly *et al*. 2014) and our results identify PAK6 as possible mediator of LRRK2 activity within these processes.

We collected additional evidence both from mouse and human brain tissues supporting a mechanism where LRRK2 is important in the activation mechanism of PAK6. LRRK2 kinase activity is not sufficient to directly activate PAK6 *in vitro* suggesting that complex cellular machinery is required. Accordingly, we found that ablation of LRRK2 causes a significant reduction of activated PAK6 in the brain and a parallel decrease in the phosphorylation levels of the PAK6 substrate LIMK1. Recently, LRRK2 was suggested to function as a scaffold, compartmentalizing protein kinase A *via* its ROC domain (Parisiadou *et al*. 2014). Together, our findings support a similar scenario where the ROC domain of LRRK2, in analogy to the Rho GTPases, re‐localizes PAK6 during signaling (Ha *et al*. 2012).

Both LRRK2 and PAK6 are expressed in human brain (Taymans *et al*. 2006; Nekrasova *et al*. 2008, Mandemakers *et al*. 2012) and deregulation of the LRRK2‐PAK6 signaling because of LRRK2 mutations may affect neuronal communication with consequent pathological outcomes. To this regard, we found that PAK6 exhibits increased autophosphorylation in G2019S and iPD brains, indicating the presence of hyperactive PAK6 in sporadic and LRRK2‐linked PD. We speculate that overactive PAK6 owing to mutant LRRK2 may result in deregulated actin cytoskeleton dynamics *via* LIMK1 with impact on neurite growth and synaptic activity. Our data may imply that overactive PAK6 owing to LRRK2 mutation is associated with increased neurite outgrowth. However, it has been reported that the G2019S induces neurite retraction (Parisiadou *et al*. 2009). While it remains unclear whether the shorter neurite phenotype linked to G2019S depends on its intrinsic higher toxicity (Greggio *et al*. 2006; Smith *et al*. 2006) or to a specific alteration of the signaling stimulating neurite outgrowth, further investigation is clearly necessary to shed light into the complex relationship among pathological LRRK2, PAK6 activation and neuronal degeneration.

In conclusion, starting from a protein array screening, our study reveals a novel functional interaction between LRRK2 and PAK6 in controlling neurite morphology and the molecular characterization of this interaction disclosed PAK6 as novel, explorable target for LRRK2‐linked PD.

## Supporting information


**Figure S1**. (a) Representative images of striatum slices co‐transduced with high titer rAAVs encoding. PAK6 and low titer LV‐eGFP to allow neurite tracing. (b) Representative images of striatum slices from LRRK2 wild‐type and knock‐out mice. transduced with rAAVs encoding PAK6 and DAB stained using anti FlagM2 antibodies.
