# Supplementary material for: Leucine‐rich repeat kinase 2 interacts with p21‐activated kinase 6 to control neurite complexity in mammalian brain
Source: J Neurochem. 2015 Oct 19;135(6):1242–56. doi: 10.1111/jnc.13369 (PMC4715492; doi:10.1111/jnc.13369)
Supplement: Supplementary file 1 — Figure S1. (a) Representative images of striatum slices co‐transduced with high titer rAAVs encoding. PAK6 and low titer LV‐eGFP to allow neurite tracing. (b) Representative images of striatum slices from LRRK2 wild‐type and knock‐out mice. transduced with rAAVs encoding PAK6 and DAB stained using anti FlagM2 antibodies. [file JNC-135-1242-s001.pdf]

Supplementary information

Title: LRRK2 interacts with PAK6 to control neurite complexity in mammalian brain.

Laura Civiero[1], Maria Daniela Cirnaru[2], Alexandra Beilina[3], Umberto Rodella[1,4], Isabella Russo[1], Elisa Belluzzi[1], Evy Lobbestael[4], Lauran Reyniers[4], Geshanthi Hondhamuni[5], Patrick A. Lewis[6,7], Chris Van den Haute[4,8], Veerle Baekelandt[4], Rina Bandopadhyay[5], Luigi Bubacco[1], Giovanni Piccoli[2], Mark R. Cookson[3,CA], Jean-Marc Taymans[4,CA,§] and Elisa Greggio[1,CA]

[1] Department of Biology, University of Padova, 35131, Padova, Italy

[2] San Raffaele Science Park and Università Vita-Salute San Raffaele, 20132 Milano, Italy

[3] Laboratory of Neurogenetics, National Institute on Aging/NIH, 20892, Bethesda, MD, USA

[4] Laboratory for Neurobiology and Gene Therapy, KU Leuven, 3000 Leuven, Belgium

[5] Reta Lila Weston Institute of Neurological Studies, Department of Molecular Neuroscience UCL, Institute of Neurology, London, WC1N 1PJ, UK

[6] School of Pharmacy, University of Reading, Whiteknights, Reading RG6 6AP, UK

[7] Department of Molecular Neuroscience, UCL Institute of Neurology, Queen Square, London, WC1N 3BG, UK

[8] Leuven Viral Vector Core, KU Leuven, 3000 Leuven, Belgium

[CA] To whom the correspondence should be addressed:

Elisa Greggio PhD, Department of Biology, University of Padova, 35131, Padova, Italy. Email: elisa.greggio@unipd.it

Jean-Marc Taymans PhD, § Current address: Jean-Pierre Aubert Research Center, UMR837, rue Polonovski - 1 place de Verdun, 59045 Lille, France. Email: jean-marc.taymans@inserm.fr

Mark R. Cookson PhD, Laboratory of Neurogenetics, National Institute on Aging/NIH, 20892, Bethesda, MD, USA. Email: cookson@mail.nih.gov

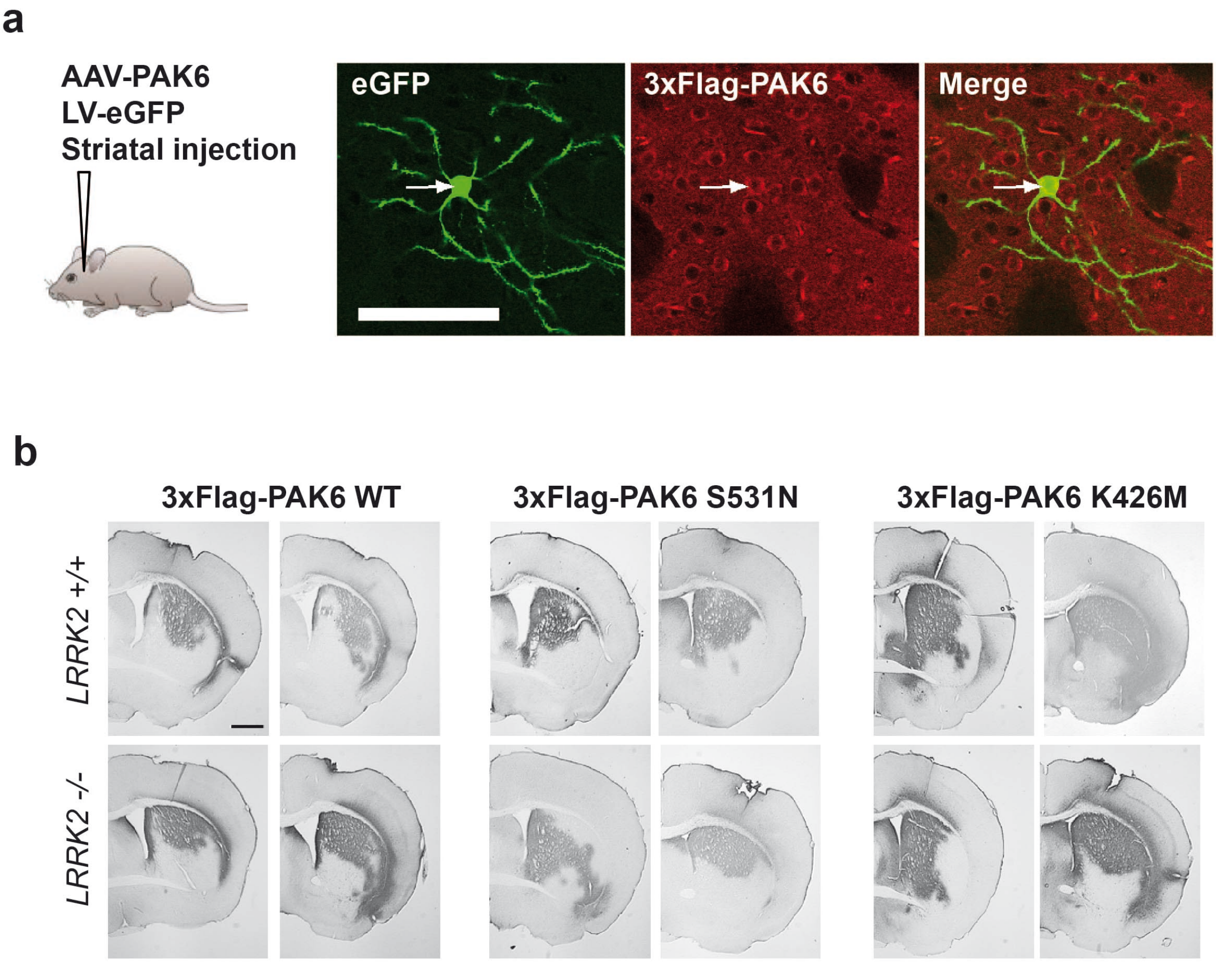

Supplementary figure 1.

a) Representative images of striatum slices co-transduced with high titer rAAVs encoding PAK6 and low titer LV-eGFP to allow neurite tracing.

b) Representative images of striatum slices from LRRK2 wild-type and knock-out mice transduced with rAAVs encoding PAK6 and DAB stained using anti FlagM2 antibodies.
